# Better Outcomes through Learning, Data, Engagement, and Research (BOLDER) – a system for improving evidence and clinical practice in low and middle income countries

**DOI:** 10.12688/f1000research.8392.1

**Published:** 2016-04-18

**Authors:** BOLDER Research Group

**Keywords:** BOLDER, clinical decision making, learning health systems, low income countries, middle income countries, Africa

## Abstract

Despite the many thousands of research studies published every year, evidence for making clinical decisions is often lacking. The main problem is that the evidence available is generated in conditions very different from those that prevail in routine clinical practice and with patients who are different. This is particularly a problem for low and middle income countries as most evidence is generated in high income countries.

A group of clinicians, researchers, and policy makers met at Bellagio in Italy to consider how more relevant evidence might be generated. One answer is to conduct more pragmatic trials—those undertaken in routine clinical practice. The group thought that this might best be achieved by developing “learning health systems” in low and middle income countries.

Learning health systems develop in communities that include clinicians, patients, researchers, improvement specialists, information technology specialists, managers, and policy makers and have a governance system that gives a voice to all those in the community. The systems focus on improving outcomes for patients, use a common dataset, and promote quality improvement and pragmatic research. Plans have been developed to create at least two learning systems in Africa.

Between 2% and 53% (median 19%) of treatments offered to patients lack substantial research to support them ^1^. A study of 16 guidelines from the American College of Cardiology and the American Heart Association found that only 314 (11%) recommendations of 2711 were supported by the highest level of evidence ^2^; and cardiovascular medicine is probably the best researched part of clinical practice.

This deficiency is even more serious in low and middle income countries because most research is conducted in high income countries and may not be applicable in low and middle income countries. Those countries have rapidly rising rates of non-communicable disease (NCD), but an analysis of the 633 systematic reviews related to NCD found that almost 90% of 8850 trials included in the reviews were from high income countries, 5% from low-middle income countries, and only 13 (0.15%) from low income countries ^3^.

At the same time as we have inadequate evidence to support many clinical decisions, clinicians are wary when we do have evidence, doubting its relevance to their local situations. In combination, these two kinds of evidence deficiency are depriving patients of access to the best treatments. How might more useful evidence be produced more efficiently? A group of clinicians, researchers, and policy makers, mostly from Africa, met in 2015 at the Bellagio Centre and developed some tentative answers.

## What is useful evidence?

Useful evidence has two components. It must be internally valid in that users of the research can be confident that its conclusions are supported by its methods and results. But it must also be externally valid, meaning that it is applicable in a wide range of circumstances, in the "real world" as opposed to the ideal world common in most clinical trials. This problem of the "applicability" of research is particularly acute for those in low and middle income countries as most research has been conducted in high income countries ^3^. Lower income countries need evidence on their own health care challenges, and they need it to be generated within their populations by their patients, clinicians, and researchers.

Studies, particularly clinical trials, may lack internal validity because they are too small, too short term, fail to remove bias, too poorly done, use surrogate outcome measures irrelevant to patients and unconvincing to clinicians, or too poorly reported. A study of 2000 randomised trials in schizophrenia found that most were not useful for making clinical decisions: studies were short (54% lasted less than six weeks), small (mean number of patients 65), and poorly reported (64% had a quality score of less than or equal to two when the maximum score was five) ^4^. Furthermore, the studies tested over 600 different interventions and used 640 different rating scales to measure outcomes, making interpretation for clinical use almost impossible ^4^.

External validity may be lacking because the patients are highly selected, excluding, for example, the old and those with multiple conditions, the research setting is not like those in which the treatment will be applied, the conditions of the research protocol are highly controlled, and patients monitored in a way that is not possible in everyday practice. Most drug trials fall into this category because they are what is required by regulators to allow drugs into the market. Furthermore, the drugs may be tested against placebo, when the question that matters to clinicians and policy makers is whether they are better than other currently used existing treatments, not only other drugs. Applicability is a particular problem in paediatrics as most studies are conducted in adults.

## More relevant research

So why not make research more relevant and - at the same time - more effective? At Bellagio our working group developed a concept called BOLDER (Better Outcomes through Learning Data and Engaging in Research: www.bolderresearch.org). One key element of BOLDER is pragmatic research. Pragmatic studies are those conducted in routine clinical practice settings, and patients are enrolled with few selection criteria in order to maintain the representativeness of the true population ^5^. In addition, the organisation of the studies should be simple, as few extra data as possible should need to be collected, and the outcome measures used should matter to those who take part in the trial, both patients and clinicians ^6, 7^. The hope is that the clinicians or policy makers will accept the results of the trial and act on them. Ideally these studies should be conducted rapidly and cheaply, avoiding the long delays and substantial costs of many trials, and be largely done by the clinicians who are the main consumers of clinical research.

## Learning health systems

Before the meeting it wasn't clear how this more useful research might be achieved, particularly in Subsaharan Africa, but during the meeting a possible answer emerged--the creation of “learning health systems.” A learning health system is one in which patients and providers work together to coproduce new knowledge and share decisions regarding best evidence ^8^. It drives discovery but is a natural outgrowth of patient care. It increases innovation, quality, and safety, and does this in real time.

Quality improvement science identifies barriers to improving health outcomes, finds ways to try and overcome them, evaluates the impact of interventions, and - if services and patients’ outcomes improve - keeps the cycle of improvement going. But the worlds of quality improvement and formal “research” rarely collide. Systems that bring these two worlds together do now exist, however, in a few places in the US and Europe. At the meeting we heard about ImproveCareNow ^9^, which began in 2007 when eight paediatric gastroenterology practices came together to improve the care of children with inflammatory bowel disease. Agreeing on an outcome measure of remission, the system established a common dataset, standardised care, and engaged patients and families. Using cycles of improvement it increased remission rates over seven years from an average of 50% to 80%. During that time it grew from eight practices to over 80 ^9^. The system then began to conduct research studies, thus becoming a true learning health system.

ImproveCareNow served as the prototype for a national, multispecialty learning health system called PEDSnet, which is now expanding to include many more hospitals and children and is conducting several pragmatic trials ^10^. It is part of a wider network, PCORnet, that includes 12 other networks like PEDSnet. PCORnet provides care to 75 million Americans and is an unsung benefit of Obamacare.

## The six components of a learning system

A successful learning system has six components.

- A community, which ideally will include clinicians, patients, researchers, improvement specialists, information technology specialists, managers, and policy makers.
- A focus on outcomes. The learning health system must produce better outcomes for patients. If it doesn't it will --and should--fold.
- A common dataset that is as simple as possible with data being entered only once. Extra data might be collected for particular studies.
- Quality improvement, which reliably applies evidence generated from research when and where patients can benefit.
- Pragmatic research
- Governance, which should ensure a voice for all those in the system, particularly patients.

## A learning system for Africa?

But could a learning health system work in Africa? The conviction of those at the meeting was that it could. Nascent platforms were identified in Kenya, Malawi, Zambia, and South Africa, and interrogation of leaders from the Kenyan and South African platforms made those at the meeting think that learning health systems could be developed in those two countries at least ^11^.

In Kenya the Wellcome KEMRI network of hospital paediatricians has developed a core dataset that is collected on every single patient who is admitted and is able to conduct research using these data. Several important randomised controlled trials have already been completed using this platform ^12, 13^.

In South Africa there is a well developed national system to incorporate current evidence based clinical guidelines into daily clinical practice in primary care. The guidelines reach tens of thousands of nurses and doctors and have improved the care of millions of patients across the entire country ^14^.

In BOLDER we are working to build on these capacities. The aim in Kenya is to develop a learning health system that can rapidly implement into daily practice across the country the evidence it gathers from pragmatic research. In South Africa we hope to build a basic electronic data platform that can be used in routine care in even the most remote facilities but can also be used to conduct research in these real world practice settings.

## Conclusion

A potential answer to the problem of inadequate evidence for clinical practice, particularly in Africa, has become clear. A learning health system will be built on networks that have already been scaled up in two countries in Africa. The systems will concentrate on improving outcomes and include all stakeholders, including patients, clinicians, and researchers. The research will happen in a context that allows it to be quickly implemented, and the aim is for the research to be pragmatic and be done quickly and cheaply. Plans are launched to make it happen.

## Note

The first draft of this article was written by RS, and there is some overlap with a blog he posted immediately after the meeting: http://blogs.bmj.com/bmj/2015/08/11/richard-smith-how-to-fill-the-void-of-evidence-for-everyday-practice/

## References

[1] Jamtvedt G Klemp M Mørland B: Responsibility and accountability for well informed health-care decisions: a global challenge. *Lancet.* 2015;386(9995):826–828. 10.1016/S0140-6736(15)60855-8 26085031

[2] Tricoci P Allen JM Kramer JM: Scientific evidence underlying the ACC/AHA clinical practice guidelines. *JAMA.* 2009;301(8):831–841. 10.1001/jama.2009.205 19244190

[3] Heneghan C Blacklock C Perera R: Evidence for non-communicable diseases: analysis of Cochrane reviews and randomised trials by World Bank classification. *BMJ Open.* 2013;3(7): pii: e003298. 10.1136/bmjopen-2013-003298 23833146 PMC3703573

[4] Thornley B Adams C: Content and quality of 2000 controlled trials in schizophrenia over 50 years. *BMJ.* 1998;317(7167):1181–4. 10.1136/bmj.317.7167.1181 9794850 PMC28699

[5] Raymond J Darsaut TE Altman DG: Pragmatic trials can be designed as optimal medical care: principles and methods of care trials. *J Clin Epidemiol.* 2014;67(10):1150–6. 10.1016/j.jclinepi.2014.04.010 25042688

[6] Tosh G Soares-Weiser K Adams CE: Pragmatic vs explanatory trials: the pragmascope tool to help measure differences in protocols of mental health randomized controlled trials. *Dialogues Clin Neurosci.* 2011;13(2):209–15. 21842618 10.31887/DCNS.2011.13.2/gtosh PMC3182001

[7] Loudon K Treweek S Sullivan F: The PRECIS-2 tool: designing trials that are fit for purpose. *BMJ.* 2015;350:h2147. 10.1136/bmj.h2147 25956159

[8] Institute of Medicine (US) Roundtable on Evidence-Based Medicine; Olsen LA Aisner D: The Learning Healthcare System: Workshop Summary. Washington (DC): National Academies Press;2007. 10.13184/eidon.39.2013.89-91 21452449

[9] Greene SM Reid RJ Larson EB: Implementing the learning health system: from concept to action. *Ann Intern Med.* 2012;157(3):207–10. 10.7326/0003-4819-157-3-201208070-00012 22868839

[10] Forrest CB Margolis P Seid M: PEDSnet: how a prototype pediatric learning health system is being expanded into a national network. *Health Aff (Millwood).* 2014;33(7):1171–7. 10.1377/hlthaff.2014.0127 25006143

[11] English M: Building Learning Networks to accelerate research and uptake of evidence to improve quality and outcomes of clinical care in low-income settings. Submitted for Publication.

[12] English M Mohammed S Ross A: A randomised, controlled trial of once daily and multi-dose daily gentamicin in young Kenyan infants. *Arch Dis Child.* 2004;89(7):665–669. 10.1136/adc.2003.032284 15210501 PMC1719980

[13] Agweyu A Gathara D Oliwa J: Oral amoxicillin versus benzyl penicillin for severe pneumonia among Kenyan children: a pragmatic randomized controlled noninferiority trial. *Clin Infect Dis.* 2015;60(8):1216–24. 10.1093/cid/ciu1166 25550349 PMC4370168

[14] Fairall L Bateman E Cornick R: Innovating to improve primary care in less developed countries: towards a global model. *BMJ Innov.* 2015;1(4):196–203. 10.1136/bmjinnov-2015-000045 26692199 PMC4680195

