# Supplementary material for: Better Outcomes through Learning, Data, Engagement, and Research (BOLDER) – a system for improving evidence and clinical practice in low and middle income countries
Source: F1000Res. 2016 Apr 18;5:693. [Version 1] doi: 10.12688/f1000research.8392.1 (PMC5089159; doi:10.12688/f1000research.8392.1)
Supplement: Peer review history

# Peer review: Referee response for version 1

**DOI:** 10.5256/f1000research.9025.r17309

**Published:** 2016-10-31

**Authors:** Lilian Dudley

**Affiliations:** 1 Division of Community Health, Department of Interdisciplinary Health Sciences (DIHS), Stellenbosch University, Stellenbosch, South Africa

**Overview**

 This opinion paper addresses important concerns about the gap between clinical evidence generation, particularly clinical trials in high income countries (HIC), their appropriateness for low and middle income country (LMIC) health systems, and the challenges of translating research evidence into practice in different settings.

 The authors propose the use of pragmatic research (trials) methods in LMIC’s within the framework of ‘learning health systems’ (LHS) as a potential solution to the current problems. The proposed approach suggests ways of conducting clinical research within ‘real world’ settings, and mechanisms by which the research evidence can be more appropriate, and facilitate implementation by practitioners and patients particularly in LMIC’s. The opinion paper generates helpful ideas and discussion on ways of reducing the disconnect between knowledge of what works in selected or ideal settings, and producing more appropriate local knowledge with the involvement of practitioners as the primary users of the knowledge.

**Frameworks of LHS, HSR and QI**

 The authors have applied components of LHS’s to the use of clinical research evidence in LMIC’s. The interpretation of LHS has not fully explained the overall concept and framework for LHS, and its application has only used a limited perspective of LHS’s in providing a framework for clinical research methods in LMIC’s.

 LHS’s seek to address concerns re external validity and use of evidence collected through trials, and is therefore an appropriate model to consider for ‘knowledge translation’. However, what the authors do not elaborate is that it does so largely through using ‘evidence’ from electronic patient information systems, and by promoting access to, interoperability and use of electronic patient records (Big Data) to provide more patient centered evidence. This ensures that evidence is linked to real patients in practice, and can be used by real health providers and patients. It benefits from the growth of ICT’s in health in HIC’s, and the extensive access to and improved quality of routine patient information in such settings. This is underpinned by financial and human resources, and infrastructure which is able to support such ICT’s for health in HIC’s.

 The authors appropriately discuss the re-conceptualisation of ways of doing research i.e. shifting from a pre-occupation with internal validity to ensuring external validity; moving from the ‘ideal’ to ‘pragmatic’ approaches; and collaborations across ‘institutions’, between researchers and practitioners and patients.  A component of LHS which could further enhance this reconceptualization is the focus on ‘harvesting’ existing data on real patients from RHIS, rather than ‘hunting’ data on ideal selected group of patients through the expensive and time consuming collection of new data with limited external validity. One of the challenges of such an approach however is that ‘Big data’ and electronic patient record systems do not exist in many LMIC’s. Many have very basic routine health information systems (RHIS), in which the quality of the data is often poor; and very few have electronic patient record systems to generate the kind of evidence needed for clinical decision making and to support a new way of doing research.

 Although the opinion piece speaks to LHS, by focusing narrowly on the potential of ‘pragmatic’ trials as a research approach it does not address fully how LHS can be used to promote the generation of evidence from different sources and its translation into practice. It would be useful to explore further the opportunities which LHS present to review the nature of evidence, and the kind of research which can be conducted i.e. sources of data, greater patient centredness, and the role of clinicians/practitioners as researchers and users of evidence in LMIC settings.

 By limiting the definition and understanding of LHS to one which simply conducts pragmatic trials rather than RCT’s, it misses the opportunity for researchers to rethink the model of research in LMIC’s, and the links between research, RHIS, and the opportunities it can create for ‘co-creation’ and use of knowledge between researchers, practitioners, patients and families to inform and transform practice. It would be useful if the authors could comment on these issues related to LHS and its application in LMIC’s, and whether any consideration was given to it in the Bellagio meeting.

 The proposal also draws on different approaches to research already practiced as part of ‘health services’ or ‘health systems’ research. The authors propose more collaborative and participative research practices, without referring to the broad and well established field of participatory action research (PAR), which facilitates ‘transformative’ research. PAR specifically aims to include users of knowledge in the research process in order to empower and build capacity of users of research (and researchers) to ensure implementation and sustainability of interventions. LHS and its application in this opinion paper, is therefore not new in identifying processes for greater involvement of users, and it would be helpful to reflect on how lessons from PAR for supporting ‘action learning’ can support such a process.

 The proposal also draws on Quality Improvement, through which evidence is implemented in collaborative and iterative processes to improve practice, and through which both researchers and practitioners learn.  The artificial divide between QI and research has diminished substantially in recent years and QI is increasingly underpinned and supported by QI research on a continuum between research and practice. The HIC projects which the authors identify are useful examples of QI supported by research. It would have been helpful if the authors could identify similar QI projects in LMIC where research is a core component of the project to illustrate that this is indeed feasible in such settings.

**Pragmatic Trials as an approach**

 In resource constrained environments such as in LMIC’s, barriers to doing RCT’s include limited financial resources, research and institutional capacity to support any form of trials. The paper does not indicate how pragmatic trials will overcome these constraints to make the kinds of evidence more easily producible and accessible in LMIC’s.

 Page 2, para 5: Although the emphasis of LHS is not on improving internal validity, the authors do raise some concerns about internal validity of clinical trials. However, the article then does not adequately address how pragmatic trials as part of LHS’s can overcome these internal validity shortcomings of RCT’s. If LHS and pragmatic trials are not addressing internal validity, perhaps this section should not receive the level of emphasis – or it needs to be addressed in the context of LHS and pragmatic trials.

 Page 2, para 7: ‘as few extra data as possible should need to be collected’ – If the LHS approach to pragmatic trials includes increased use of routine data, it is important to discuss whether suitable data is available, and indicate how pragmatic trials will overcome the limitations of routine data in many LMIC’s, as well as the lack of trust in the data by clinicians.

 Page 2, para 7: The emphasis on clinicians doing the studies may not fully take the reality of LMICs into account. Clinicians are a scarce resource in most LMIC’s and tend to be overburdened with clinical work with little time to undertake additional research activities. Many also do not have the necessary training and research skills to participate in such studies. It would therefore be useful for the opinion piece to indicate how and why it will be easier under these circumstances for clinicians to do pragmatic trials as opposed to other forms of trials or research; and how LHS will contribute to facilitating this and building capacity to undertake these trials.

 Page 2, para 8, proposes a more collaborative and participative research process, but does not reference the extensive experience and lessons of PAR – which precisely aims to not only undertake research under real world conditions, and involve participants and stakeholders and create a learning process for both. Much could be learnt by pragmatic triallists by considering PAR approaches which already describe ways of engaging and joint learning, but also highlight some of the limitations.

 Page 2, para 9: There is a continuum from QI practice to QI research which is increasingly recognised and practiced. Unfortunately the only examples provided of QI research are from HIC projects, and it would have been useful to identify examples of QI research in LMIC’s. It does not help the authors’ argument that LHS need to be local in LMIC, if there is little evidence of that happening.

 Page 3, para 2: Patient centredness is a key element of LHS which receives little focus in the paper. In HICs’ an important component of using electronic patient data is that the data is linked directly to real patients, and can include their experiences and assessments of outcomes. It would be useful for the opinion paper to indicate how this aspect can be used and applied in LMIC’s as part of pragmatic research.

 Page 3, para 4 – the database of clinical guidelines in SA, although a step in the right direction, does not really encompass the scope of activities proposed in the paper or typical of a LHS. It would be helpful if the authors could indicate how this links to the earlier suggestions of pragmatic trials, and the involvement of clinicians in research.

 Page 3, para 5: The suggestion that new electronic data collection systems for research be established is also contrary to earlier suggestions of limiting the requirement to collect new data. There have been several initiatives to establish electronic patient records in South Africa and the authors should rather investigate what has already been developed and implemented.  LMIC’s are littered with projects which have set up independent data collection systems instead of strengthening the routine information systems, and this needs to be approached with caution.

 Page 3, para 6: Before proposing this as  a ‘clear’ solution, it would be useful to have more engagement with clinicians, policy makers, patients, ICT personnel and to look at current practices in LMIC’s in order to develop a clearer framework for how LHS’s and pragmatic research can support the use of evidence in LMIC’s.

I have read this submission. I believe that I have an appropriate level of expertise to confirm that it is of an acceptable scientific standard.

# Peer review: Referee response for version 1

**DOI:** 10.5256/f1000research.9025.r14218

**Published:** 2016-07-27

**Authors:** Gillian Mann

**Affiliations:** 1 Department for International Development, London, UK

**Please note that I am writing in my personal capacity; this is not an official response from the Department for International Development**

 This is a helpful contribution to the health research agenda. For some time researchers have been asking how to have research taken into policy - this goes some way to answer questions of why that does not often happen. 

 It would help if more examples outside the specific areas of clinical practice were given. For example research may not only be on one narrow interest topic (though this clearly has validity), but may be on broader approaches to patient care - e.g. improving outreach services in community based care, reaching neglected patient groups with services that already work for others, changing broad clinical protocols to improve patient pathways to care.

 It would also help to highlight some of the contextual reasons why research has failed to be taken up in the past - e.g. cost of new technologies (not just cost effectiveness), a lack of understanding of what is needed to make new technologies work (e.g. staffing skills, numbers, distribution; consistent availability of water or electricity etc). Previous work relating to the types of evidence required has previously been undertaken ^1^.

 Two phrases are used rather interchangeably throughout: "Learning Health Systems" and "Learning Systems".  I would argue that the latter is more applicable in this context.  A "learning health system" implies that the broad health system is learning, whereas this article refers to establishing systems to learn to improve clinical practice in particular, which may only relate to parts of the health system, circumscribed by geography, field or research, or some other criteria. While this may evolve into a broader learning health system, the term is rather grander than the scope at this time.

I have read this submission. I believe that I have an appropriate level of expertise to confirm that it is of an acceptable scientific standard.

## References

[1] MannGSquireSBBissellKEliseevPDu ToitEHesselingANicolMDetjenAKritskiA: Beyond accuracy: creating a comprehensive evidence base for TB diagnostic tools. *Int J Tuberc Lung Dis*.2010;14(12) :1518-24 21144235
